# The long-term mental health benefits of exercise training for physical education students: a comprehensive review of neurobiological, psychological, and social effects

**DOI:** 10.3389/fpsyt.2025.1678367

**Published:** 2025-09-16

**Authors:** Yuxin Ma, Somna Mumtaz

**Affiliations:** ^1^ Zhengzhou Urban Construction Vocational College, Zhengzhou, Henan, China; ^2^ Department of Applied Psychology, Riphah International University, Islamabad, Pakistan

**Keywords:** exercise training, mental health, physical education students, neuroplasticity, stress resilience, social connectedness, mindfulness, academic performance

## Abstract

This systematic review examines the long-term effects of exercise training interventions on the mental health of physical education (PE) students, integrating neurobiological, psychological, cognitive, and social perspectives. Evidence indicates that structured exercise programs, including aerobic training, resistance exercise, and mindfulness-based practices, enhance mental well-being through multiple pathways. Aerobic activity elevates brain-derived neurotrophic factor (BDNF) and serotonin, improving mood and stress resilience, while resistance training fosters self-efficacy and emotional regulation. Team sports and group-based interventions mitigate social isolation by strengthening peer bonds, a critical factor in preventing depression and anxiety. Cognitive benefits, such as enhanced memory and academic performance, are linked to exercise-induced neurogenesis and increased cerebral blood flow. However, gaps persist in longitudinal research (>5 years), standardized protocols, and cultural adaptations. Practical recommendations for universities and coaches include integrating mental health monitoring, balancing training intensity to prevent burnout, incorporating mindfulness practices, and promoting peer support networks. By adopting a holistic approach that combines physiological and psychosocial strategies, PE programs can optimize both mental health and academic outcomes. This synthesis underscores the need for evidence-based, multimodal interventions tailored to the unique demands of student-athletes, ultimately supporting their development as high-performing and psychologically resilient individuals.

## 1 Introduction

Mental health challenges among university students have become a critical public health concern, with increasing rates of anxiety, depression, and stress-related disorders (1). The severity of this issue is further underscored by rising suicide rates, particularly among vulnerable demographics, highlighting an urgent need for effective, accessible, and multifaceted intervention strategies that extend beyond traditional talk therapy (2). While physical activity is widely recognized for its psychological benefits, students majoring in physical education (PE) face unique stressors, including performance pressure, injury risks, and academic-athletic balance, which may undermine these advantages (3). Paradoxically, despite their high fitness levels, emerging evidence suggests that PE students experience comparable, or even higher, levels of psychological distress than their non-PE peers (4). This raises important questions about whether structured exercise interventions can provide long-term mental health resilience or if additional psychological support is needed.

The existing literature predominantly focuses on the short-term effects of exercise on mental health, leaving a gap in understanding how sustained training interventions influence psychological well-being over time (5). While acute exercise boosts mood through endorphin release and stress reduction, the long-term neurobiological adaptations, such as increased brain-derived neurotrophic factor (BDNF) and enhanced stress resilience, remain understudied in PE students (6). Furthermore, most research has examined general student populations, neglecting the unique pressures faced by those in competitive sports and PE programs. This review seeks to address this gap by analyzing longitudinal studies on exercise interventions and their lasting mental health effects in this specific demographic.

The primary objective of this study is to critically evaluate the long-term (≥6 months) impact of structured exercise training on the mental health of PE students, focusing on anxiety, depression, stress resilience, and cognitive function. Additionally, this review aims to identify optimal exercise modalities (e.g., aerobic, resistance, or mindfulness-based training) and intensities that maximize psychological benefits while minimizing risks such as overtraining syndrome (7). By synthesizing findings from clinical trials, cohort studies, and meta-analyses, this article provides evidence-based recommendations for universities and coaches to enhance mental health support within PE curricula. This study is important for several reasons: (1) it challenges the assumption that PE students are inherently protected from mental health issues due to their active lifestyles; (2) it highlights the need for tailored interventions that address both physical and psychological demands; and (3) it informs policy changes in academic and athletic programs to promote sustainable well-being. Given the rising prevalence of mental health disorders among students, this review contributes novel insights into how long-term exercise regimens can be optimized to foster resilience in future sports professionals.

## 2 Methodology of the study

**Figure 1:**
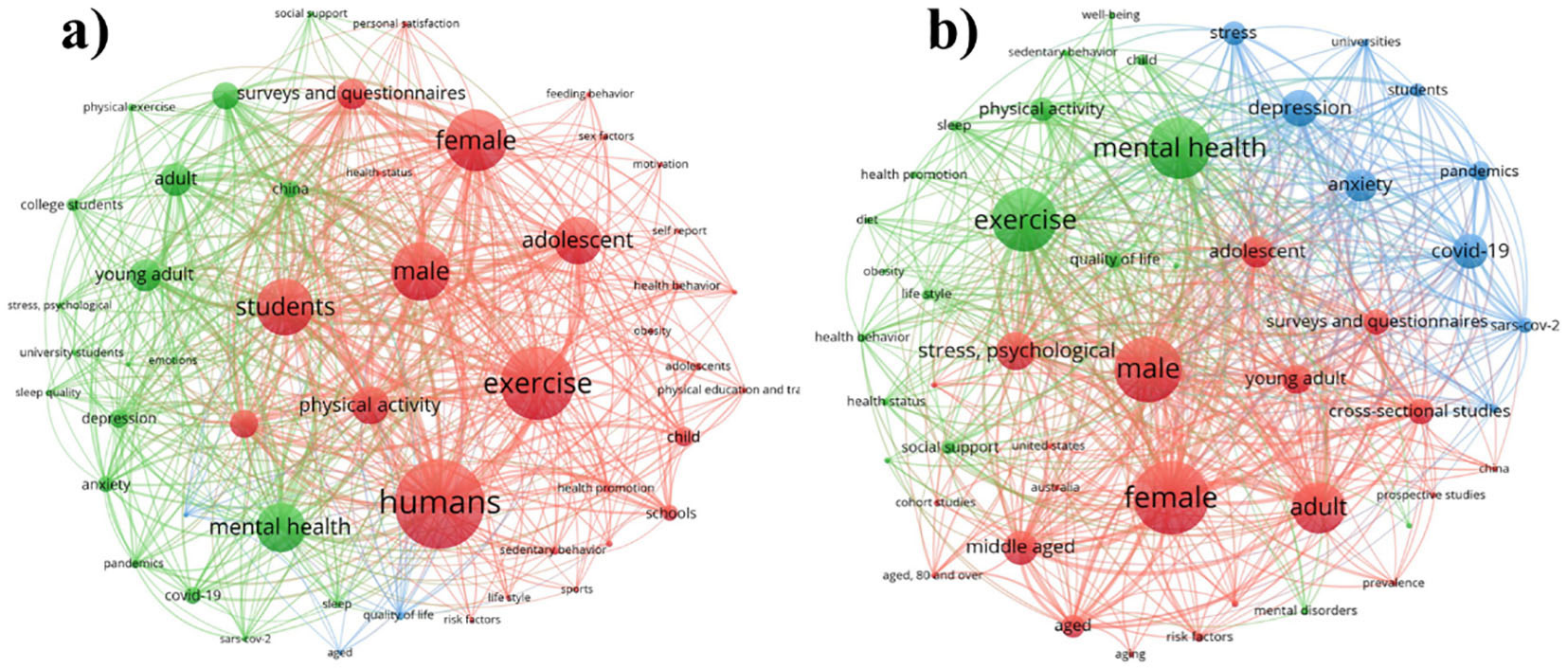
Methodological approach for conducted this study, **(a)** 1^st^ round of search, **(b)** 2^nd^ round of search using Google Scholar, Web of Science, ResearchGate, PubMed, Scopus and ScienceDirect as a search sources.

In order to carry out this study, we searched the bibliographic information using different keywords in different sites, including Google Scholar, Web of Science, ResearchGate, PubMed, Scopus and ScienceDirect. Two set of keywords were searched, including exercise training, mental health, physical education students, adolescents, and personal satisfaction as shown in (**Figure 1a**), the figure was prepared through VOSviewer and co-occurrence network was prepared based on the correlation. Following the literatures search, we trimmed the review articles from these studies and focused on the research articles. Around 2000 keywords were extracted by the VOSviewer, and were reduced to 50 keywords, prior to perform the correlation analysis. All the keywords searched were divided into three main clusters, where the 1^st^ cluster was comprised mainly of human, male, female, students, physical activity, adolescent, and other. The 2^nd^ cluster was mainly comprised of adult, mental health, depression, anxiety, emotions, and other, while the 3^rd^ cluster included quality of life, and aged. In a second round of search, we searched main keywords, including physical education students, mental health, social connectedness, exercise training and stress. The results showed number of articles on the keywords, and we further delve deep into the research articles after trimming the review articles from them. The analysis was done via VOSviewer and around 391 keywords were suggested, which was further refined into 50 keywords, and the co-occurrence network results can be seen in **Figure 1b**. According to VOSviewer, 3 clusters were suggested, including 1^st^ cluster was comprised of male, female students, adolescent, adult, aged, stressed-psychology, while 2^nd^ cluster was comprised of exercise, physical activity impact on sleep, lifestyle, health behavior, social support and well-being. The 3^rd^ cluster was mainly consisted of depression, anxiety, and stress. Following the search, each article was carefully read, and a conclusion was made on each article and was combined in a shape of this review article.

## 3 Mental health challenges among physical education students

PE students encounter several stressors that may impact their psychological well-being:

### 3.1 Performance anxiety (fear of failure in sports and academics)

**Figure 2:**
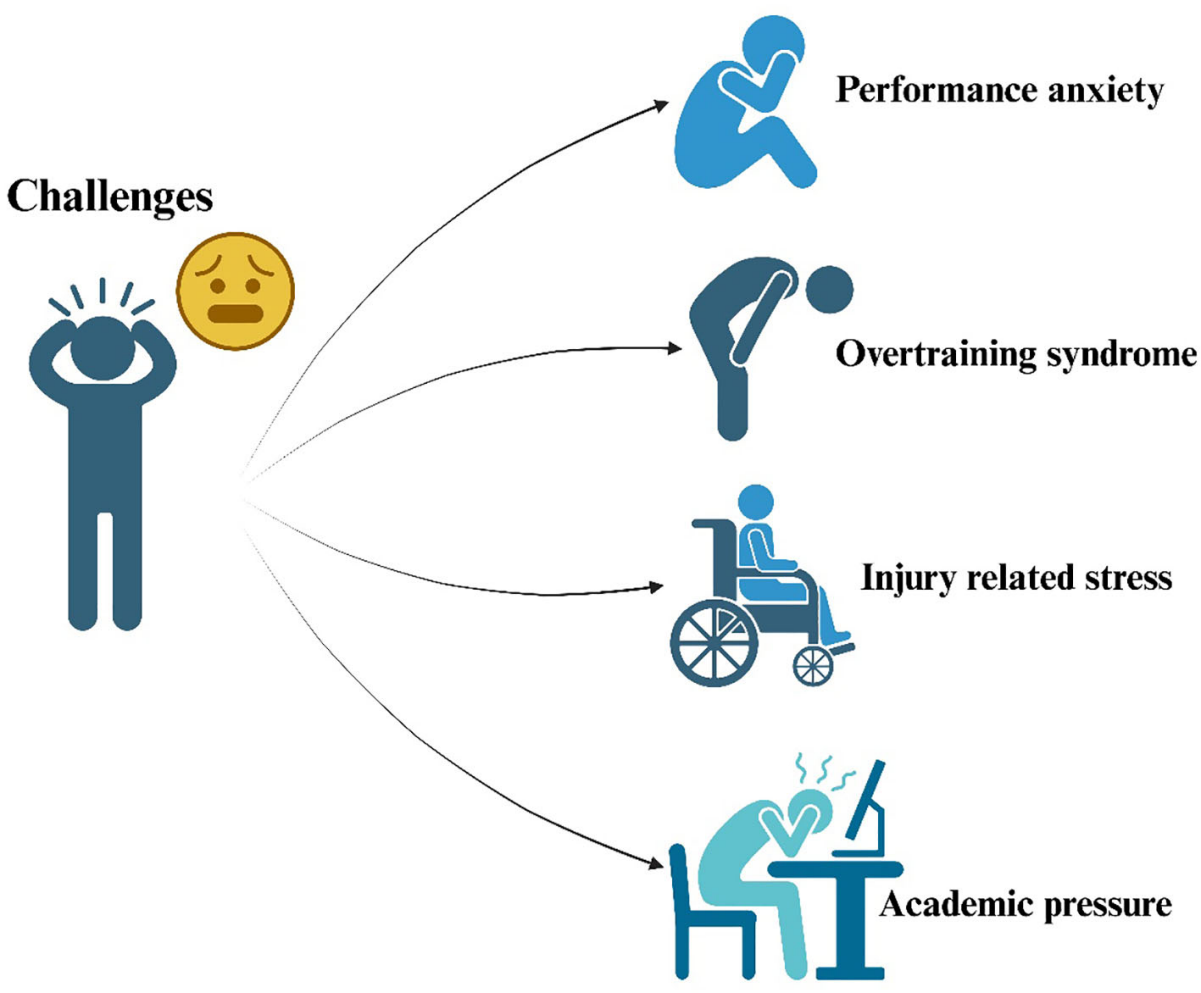
Physical education students facing mental health challenges in different ways.

Physical education (PE) students face a unique set of psychological stressors that distinguish them from their non-athlete peers (**Figure 2**; **Table 1**). One of the most prevalent issues is performance anxiety, which stems from the dual pressure of excelling in both sports and academics (8). The fear of failure in competitions, coupled with academic expectations, can lead to chronic stress, burnout, and even depressive symptoms (9). Studies suggest that nearly 30-35% of elite student-athletes experience clinically relevant anxiety levels, significantly higher than the general student population (10). This phenomenon highlights the paradoxical reality that despite their high physical fitness, PE students remain vulnerable to mental health struggles due to the intense demands of their training and education. Another critical challenge is overtraining syndrome, which arises from excessive physical exertion without adequate recovery (7). Unlike recreational exercisers, PE students often follow rigorous training schedules, increasing their risk of physical exhaustion, mood disturbances, and decreased motivation (11). Research indicates that prolonged overtraining can elevate cortisol levels, impair sleep quality, and contribute to emotional instability (12). These physiological disruptions may exacerbate pre-existing mental health conditions, creating a vicious cycle of declining performance and worsening psychological well-being.

**Table 1:** Stress factors and the associated key systems and risks affected by the lack or over-training of physical exercise and the interventions needed.

Stress factor	Prevalence (%)	Key symptoms	Performance impact	Associated risks	Coping mechanisms	Long-term effects	Gender differences	Support systems	Intervention needs
Performance Anxiety	30-35%	Fear of failure, chronic stress	Decreased focus, burnout	Depression, emotional instability	Mindfulness, goal-setting	Persistent anxiety disorders	Higher in females	Coaching support	Stress management programs
Overtraining Syndrome	Up to 60%	Fatigue, irritability, mood disturbances	Reduced athletic output	Elevated cortisol, sleep disruption	Periodized training, rest periods	Chronic fatigue syndrome	More common in males	HRV monitoring	Load management
Injury-Related Stress	30%	Depression, identity crises	Delayed recovery, reinjury	Substance use, social withdrawal	Psychological rehab, peer support	Career termination, PTSD	Equal prevalence	Counseling services	Holistic rehab programs
Academic-Athletic Imbalance	40%	Chronic stress, sleep deprivation	Lower GPA, cognitive fatigue	Burnout, social isolation	Time management, flexibility	Dropout, mental health decline	Females report higher stress	Academic advisors	Integrated scheduling
Social Isolation	25-35%	Loneliness, reduced social support	Decreased motivation	Depression, anxiety	Team-building activities	Long-term social withdrawal	Higher in individual athletes	Peer mentorship	Group-based interventions
Sleep Disturbances	45-50%	Insomnia, poor sleep quality	Impaired recovery, cognitive decline	Immune dysfunction	Sleep hygiene education	Chronic sleep disorders	No significant difference	Sleep clinics	Restorative sleep programs
Identity Crises	20-25%	Loss of self-worth, confusion	Poor performance, lack of direction	Substance abuse	Career counseling	Long-term identity issues	Higher in elite athletes	Life skills workshops	Transition programs
Financial Stress	15-20%	Anxiety about scholarships, future	Distraction, reduced focus	Poor academic performance	Financial literacy programs	Long-term financial insecurity	Higher in low-income students	Scholarships, grants	Financial planning support
Perfectionism	30-40%	Unrealistic standards, self-criticism	Burnout, avoidance behaviors	Eating disorders, depression	Cognitive-behavioral therapy	Chronic mental health issues	Higher in females	Mental health coaching	Resilience training
Fear of Failure	35-45%	Avoidance, procrastination	Underperformance, withdrawal	Anxiety disorders	Growth mindset training	Persistent avoidance behaviors	No significant difference	Performance psychologists	Confidence-building programs

### 3.2 Overtraining syndrome (leading to burnout and mood disturbances)

Physical education (PE) students face significant mental health challenges stemming from the rigorous demands of their academic and athletic pursuits. One prominent stressor is overtraining syndrome, which occurs when excessive physical training is not balanced with adequate recovery, leading to burnout, mood disturbances, and decreased performance (7). Studies indicate that up to 60% of student-athletes experience symptoms of overtraining, including chronic fatigue, irritability, and depression (12). The physiological stress of prolonged intense exercise elevates cortisol levels, disrupts sleep patterns, and impairs cognitive function, creating a vicious cycle that exacerbates mental health issues (11). Furthermore, the pressure to maintain peak performance often discourages students from seeking help, perpetuating a culture of silence around mental health struggles (3). In addition to overtraining, PE students frequently grapple with academic-athletic imbalance, where the competing demands of coursework and training schedules lead to chronic stress and diminished well-being (13). Research shows that nearly 40% of student-athletes report significant difficulties managing academic deadlines alongside athletic commitments, often sacrificing sleep and social connections to meet expectations. This imbalance is particularly detrimental as it not only increases the risk of anxiety and depression but also reduces overall life satisfaction (14). The lack of time for adequate recovery and social interaction further isolates students, compounding feelings of loneliness and stress (15). These findings underscore the need for institutional support systems that address both the physical and psychological demands unique to PE students, promoting a more sustainable approach to their dual roles as athletes and scholars.

### 3.3 Injury-related stress (fear of career disruption)

Physical education students face significant psychological stressors related to sports injuries that extend beyond physical pain. Injury-related stress, particularly the fear of career disruption, represents a major mental health challenge for these students (15). Research indicates that injured student-athletes exhibit 2–4 times higher rates of depressive symptoms compared to their non-injured peers, with many experiencing identity crises and anxiety about their athletic future (16). The psychological impact is particularly severe for those specializing in a single sport, as injuries may threaten their entire professional trajectory (17). Moreover, the pressure to return to play prematurely - often driven by academic timelines or coaching demands - can exacerbate mental health symptoms and increase reinjury risk (3). This creates a vicious cycle where physical recovery is compromised by psychological distress, potentially leading to longer-term mental health consequences. The psychological response to injury involves complex interactions between biological, psychological, and social factors (18). Studies show that approximately 30% of injured student-athletes develop clinically relevant levels of anxiety or depression during rehabilitation (10). The loss of athletic identity, social connections, and daily structure following injury contributes significantly to emotional distress (19). Furthermore, inadequate psychological support during rehabilitation often leads to poor coping strategies, including substance use or training avoidance (8). These findings highlight the need for comprehensive mental health support systems within physical education programs to address injury-related psychological distress and facilitate holistic recovery.

**Figure 3:**
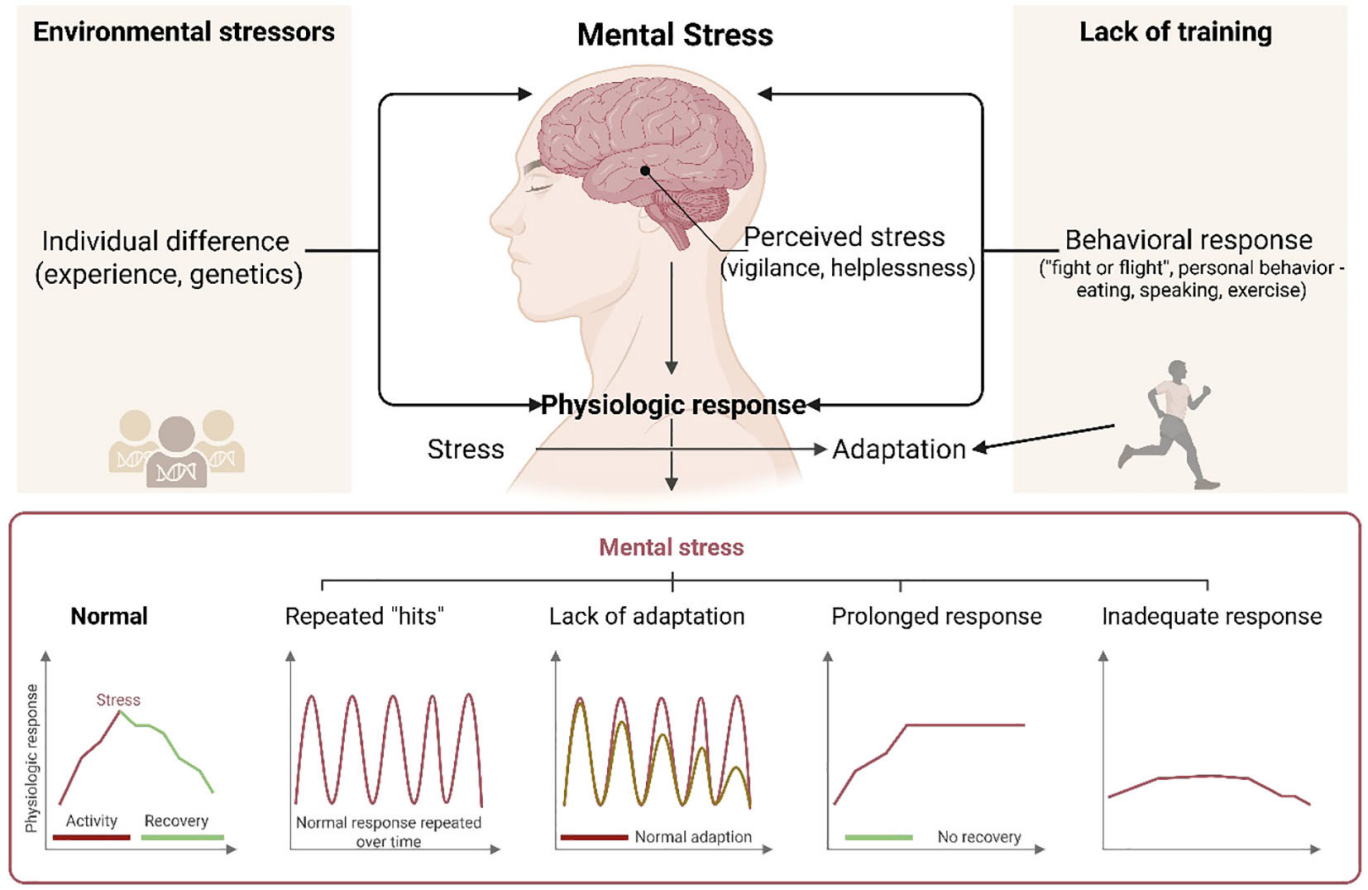
Consequences of mental health due to environmental stressors, and their change via physical exercise.

Injury-related stress further compounds mental health risks in this population. For PE students, injuries are not just physical setbacks but also threats to career aspirations, leading to heightened anxiety about rehabilitation and future performance (15). A longitudinal study found that injured athletes exhibited significantly higher depression scores compared to non-injured peers, with some experiencing post-injury identity crises (16). Additionally, the pressure to return to play prematurely, often driven by academic deadlines or coaching demands, can prolong recovery and increase susceptibility to re-injury, further impacting mental health (3). Finally, academic-athletic imbalance poses a significant psychological burden. PE students must juggle demanding training regimens with coursework, leaving little time for rest or social activities (13). This imbalance can lead to chronic stress, social isolation, and decreased life satisfaction (14). Surveys reveal that nearly 40% of student-athletes report difficulties managing academic and athletic commitments, with many sacrificing sleep or social interactions to meet expectations. Without proper support systems, these cumulative stressors may contribute to long-term mental health deterioration, underscoring the need for targeted interventions in PE programs (**Figure 3**).

### 3.4 Academic pressure (balancing coursework and athletic commitments)

Physical education students face unique academic pressures that significantly impact their psychological well-being as they attempt to balance rigorous coursework with demanding athletic commitments (13). Research indicates that approximately 40% of student-athletes report severe difficulties managing academic responsibilities alongside training schedules, often sacrificing sleep and social activities to meet expectations. This chronic time pressure leads to elevated stress levels, with studies showing student-athletes experience 20-30% higher perceived stress compared to non-athlete peers (14). The dual role creates a constant state of role conflict, where academic deadlines compete with athletic obligations, resulting in decreased life satisfaction and increased risk of burnout (9). Furthermore, the physical exhaustion from training often impairs cognitive function, making academic performance more challenging and creating a vicious cycle of stress and underachievement (12). The academic-athletic balance challenge is particularly acute during competitive seasons when training demands peak (20). Many PE students report feeling inadequately supported by academic institutions, with inflexible scheduling and limited understanding from faculty exacerbating their stress (15). This institutional mismatch contributes to mental health concerns, with surveys revealing that 25-35% of student-athletes experience clinically significant anxiety or depression symptoms (8). The pressure to maintain athletic scholarships while achieving academic eligibility creates additional performance anxiety, particularly for students from disadvantaged backgrounds (10). Without proper support systems, these cumulative academic pressures can lead to emotional exhaustion, decreased motivation, and in severe cases, premature athletic career termination (3). Despite their fitness levels, studies indicate elevated rates of anxiety and depression compared to non-PE peers, suggesting that physical activity alone may not suffice as a mental health buffer.

## 4 Exercise training interventions and mental health: mechanisms of action

Exercise influences mental health through multiple interconnected biological and psychological pathways as well as their cognitive benefits (**Table 2**).

**Table 2:** Mechanisms and key biomarkers release due to exercise modality and their subsequent impact on cognitive, emotional, social, long-term adaptation and population-specific effects.

Mechanism	Key biomarkers	Exercise modality	Duration for effects	Cognitive benefits	Emotional benefits	Long-term adaptations	Population-specific effects	References
BDNF Elevation	Increased BDNF, serotonin	Aerobic exercise	≥30 mins, 3x/week	Improved memory	Reduced depression	Hippocampal growth	Greater in PE students	(21)
Serotonergic Modulation	Tryptophan hydroxylase	Aerobic/resistance	6–8 weeks	Enhanced mood stability	Reduced anxiety	Stable serotonin levels	More pronounced in females	(22)
Endorphin Release	Beta-endorphins	High-intensity exercise	Immediate	Euphoria, pain relief	Stress reduction	Temporary	Greater in endurance athletes	(23, 24)
Neurogenesis	Hippocampal volume	Aerobic exercise	6–12 months	Better learning, memory	Emotional resilience	Long-term structural changes	Youth show faster adaptation	(25)
HPA Axis Regulation	Cortisol reduction	Yoga, mindfulness	8–12 weeks	Improved stress response	Lower perceived stress	Sustained stress resilience	Effective for high-stress students	(26)
Self-Efficacy	Perceived competence	Resistance training	3–6 months	Goal achievement	Confidence, motivation	Long-term behavioral changes	Greater in novice exercisers	(27)
Social Bonding	Oxytocin release	Team sports	Ongoing	Shared goal pursuit	Reduced loneliness	Lifelong social skills	Most beneficial in team sports	(28)
Cognitive Function	Cerebral blood flow	Aerobic exercise	30–45 mins, 3-5x/week	Enhanced attention, GPA	Reduced mental fatigue	Improved academic performance	Stronger in adolescents	(29)
Emotional Regulation	Heart rate variability	Tai chi, yoga	6–12 weeks	Better decision-making	Reduced emotional reactivity	Sustained emotional control	More effective in males	(30)
Stress Resilience	Prefrontal cortex activation	Mindfulness-based exercise	9–12 months	Improved focus	Lower anxiety relapse	Long-term neural adaptation	Beneficial for all students	(31)

### 4.1 Neurobiological effects

Exercise exerts profound neurobiological effects that enhance mental health through multiple molecular pathways. Regular physical activity significantly increases brain-derived neurotrophic factor (BDNF) expression, particularly in the hippocampus, promoting neurogenesis and synaptic plasticity, critical processes for mood regulation and cognitive function (32). The meta-analysis by Dinoff et al. found that exercise training increases resting peripheral BDNF levels, with aerobic training showing a significant increase of 31.0%. Resistance training did not significantly affect BDNF levels. The studies included in the analysis were not limited to students but included a general population. The findings suggest that aerobic exercise can enhance BDNF, which is linked to cognitive and mood benefits. (33). Concurrently, exercise modulates monoamine systems, elevating synaptic serotonin availability through increased tryptophan hydroxylase activity and reduced serotonin reuptake (34). This serotonergic enhancement, coupled with exercise-induced endorphin release from the pituitary gland and hypothalamus, creates a natural analgesic and euphoric effect that reduces anxiety and improves affective states (35). These neurochemical adaptations are particularly relevant for physical education students, as they may counteract the psychological stressors inherent in competitive athletic environments.

**Figure 4:**
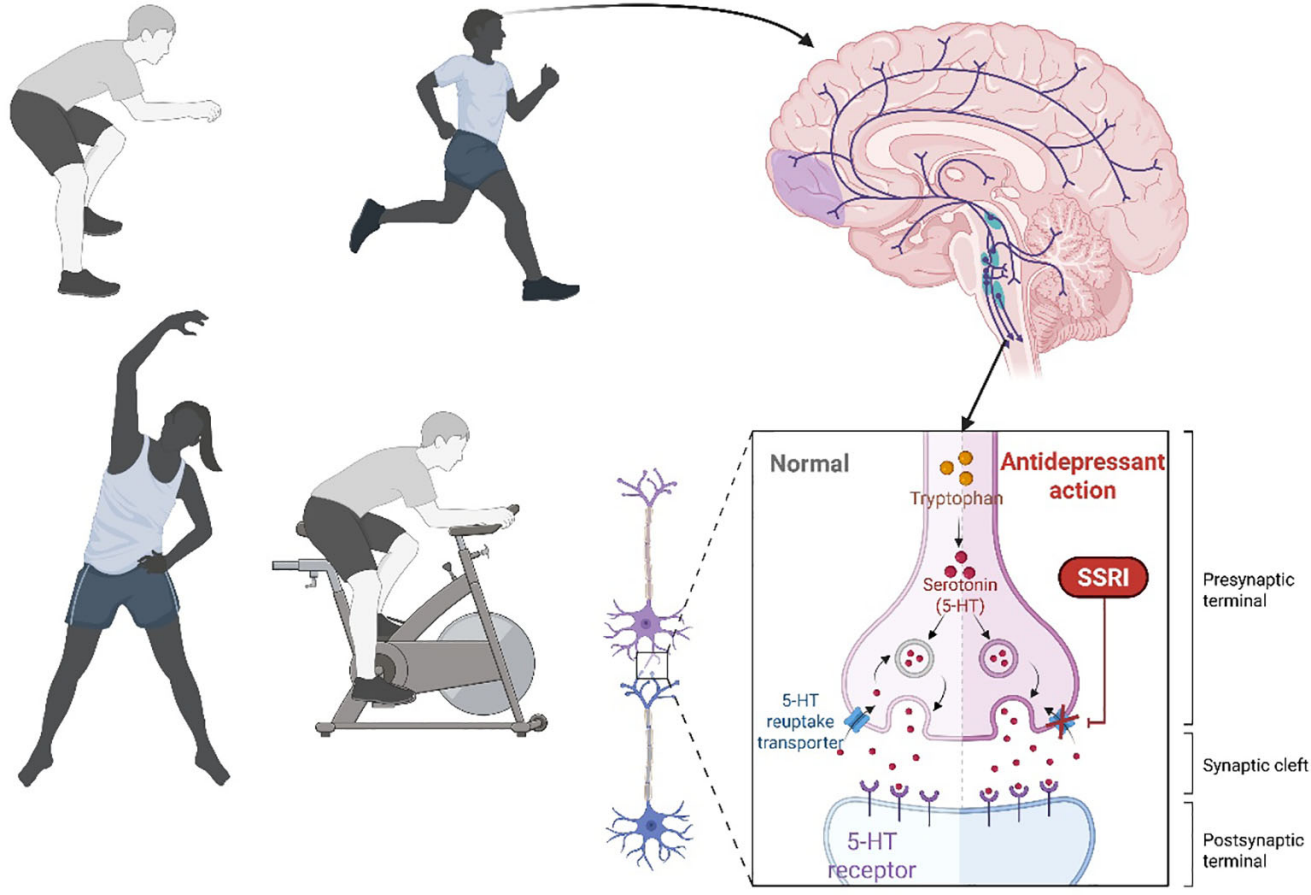
Mechanisms of induction of serotonin via physical exercise and reduction of stress.

The temporal dynamics of these neurobiological changes reveal dose-response relationships critical for intervention design. Aerobic exercise at 60-80% of maximum heart rate for ≥30 minutes appears optimal for BDNF elevation, with effects peaking immediately post-exercise and sustaining for several hours (36). Serotonergic changes follow similar kinetics but require regular training over weeks to establish stable improvements in mood regulation (37). Endorphin release exhibits acute spikes during high-intensity exercise (>75% VO_2_max), contributing to the immediate “runner’s high” phenomenon (35). Importantly, these mechanisms interact synergistically, BDNF enhances serotonin receptor sensitivity (**Figure 4**), while endorphins modulate the HPA axis, collectively producing antidepressant and anxiolytic effects (38). For PE students, structured interventions incorporating these intensity-duration parameters can maximize mental health benefits while aligning with their training regimens. While physical exercise training is widely recognized for its benefits, there are potential negative aspects that should be considered, including high-intensity exercise can lead to a temporary increase in stress hormones, which might negatively affect the brain’s neurochemistry (39), overtraining can result in a decrease in brain-derived neurotrophic factor (BDNF) levels, potentially impairing neuroplasticity and mood regulation, intensive physical activity may cause oxidative stress, which could harm brain cells if not balanced with adequate recovery, and in some individuals, exercise might trigger migraines due to the rise in serum calcitonin gene-related peptide (CGRP) levels. Therefore, future research should investigate the optimal intensity and duration of exercise to prevent these adverse effects and further explore the mechanisms behind exercise-induced migraines.

### 4.2 Psychological mechanisms

Beyond its neurobiological effects, exercise training enhances mental health through several key psychological mechanisms. Regular participation in structured physical activity fosters enhanced self-efficacy - the belief in one’s ability to successfully execute behaviors required to produce specific performance attainments (40). As individuals master progressively challenging exercise tasks, they develop greater confidence in their physical capabilities, which generalizes to improved perceived competence in other life domains (41). This is particularly relevant for physical education students, as the mastery experiences gained through sport-specific training may buffer against academic stress and performance anxiety. Research demonstrates that exercise-induced improvements in self-efficacy mediate up to 30% of the antidepressant effects of physical activity (42), highlighting its importance as a psychological mechanism. Team-based exercise training provides unique stress coping benefits through both cognitive and behavioral pathways. Group sports and cooperative training environments teach adaptive coping strategies such as problem-focused engagement, emotional regulation, and cognitive restructuring (43). The repetitive exposure to manageable physical stressors during training enhances physiological stress tolerance while simultaneously building psychological resilience - a process known as cross-stressor adaptation (44). For student-athletes, these adaptations are particularly valuable as they translate to improved handling of academic pressures and competitive demands. Studies show that team-sport participants exhibit more effective stress appraisal and coping responses compared to individual exercisers (45), suggesting the social context of training amplifies these psychological benefits.

The social support inherent in team-based training represents a third critical psychological mechanism. Shared physical activity fosters strong interpersonal bonds through mutual encouragement, cooperative goal pursuit, and collective achievement experiences (46). This social connectedness fulfills fundamental psychological needs for relatedness and belonging, which are protective factors against depression and anxiety (47). Among physical education students, team training environments provide a crucial support network that buffers against the isolation often experienced during periods of intense academic and athletic pressure. Meta-analytic evidence indicates that social support accounts for approximately 25% of the mental health benefits derived from group exercise programs (48). Furthermore, the accountability and motivational aspects of team dynamics enhance exercise adherence, creating a positive feedback loop that sustains both physical and psychological benefits over time (49). However, rigorous training schedules can lead to increased anxiety and stress, especially among competitive athletes, the pressure to perform can result in performance anxiety and fear of failure, which might exacerbate mental health issues, injuries sustained during exercise can lead to depression and anxiety, impacting an individual’s psychological well-being, and the fear of career disruption due to sports injuries can cause significant psychological stress, particularly for those specializing in a single sport (50). Therefore, further studies are needed to develop effective psychological support systems for athletes and to understand how to mitigate the psychological impact of injuries and performance pressure.

### 4.3 Cognitive benefits

Exercise training induces significant cognitive benefits through two primary physiological mechanisms: enhanced cerebral circulation and stimulated neurogenesis. Aerobic exercise increases cerebral blood flow by 15-25% during activity, with sustained improvements in baseline perfusion persisting for several hours post-exercise (51). This hemodynamic response delivers greater oxygen and nutrient supply to prefrontal cortical regions responsible for executive functions, including attention regulation, working memory, and cognitive flexibility (52). Studies using functional MRI demonstrate that physically active students exhibit more efficient neural activation patterns during cognitive tasks, requiring less prefrontal recruitment for equivalent academic performance (53). The vascular benefits are complemented by exercise-induced neurogenesis in the hippocampus, where new neuron formation increases by 30-40% following 6–8 weeks of regular aerobic training (54). These structural adaptations enhance memory consolidation and information processing speed, directly translating to improved academic outcomes.

The synergistic effects of improved circulation and neurogenesis create optimal conditions for learning and cognitive performance. Enhanced hippocampal neurogenesis facilitates faster encoding of new information, while increased prefrontal perfusion supports sustained attention during complex academic tasks (55). Meta-analytic evidence indicates that students engaging in regular physical activity demonstrate 10-15% better performance on standardized tests of mathematics and reading comprehension compared to sedentary peers (56). These cognitive benefits follow a dose-response relationship, with maximal effects observed at moderate-intensity exercise (40-60% VO2max) performed for 30–45 minutes, 3–5 times weekly (57). For physical education students, these mechanisms are particularly salient as they suggest that sport training may confer dual benefits - improving both athletic performance and academic achievement through shared neurobiological pathways.

Emerging evidence suggests that structured exercise interventions yield more substantial and enduring mental health benefits compared to unstructured physical activity. Structured programs incorporating resistance training, aerobic exercise, or mind-body practices like yoga provide systematic physiological and psychological stimuli that promote neuroplasticity and stress resilience (58). A meta-analysis of 33 clinical trials demonstrated that supervised, periodized exercise programs produced 28% greater reductions in depressive symptoms compared to unstructured activity, with effects persisting for up to 12 months post-intervention (59). The superior efficacy of structured approaches appears related to their ability to maintain optimal exercise dosage (intensity, duration, and frequency) while progressively challenging participants through periodization - a key factor in sustaining neurobiological adaptations (6). For physical education students, structured interventions may be particularly valuable as they align with athletic training principles while addressing mental health needs. The mechanistic advantages of structured interventions operate through multiple pathways. Aerobic training protocols maintaining 60-80% of heart rate reserve consistently elevate BDNF levels by 18-25%, whereas unstructured activity shows more variable effects (33). Resistance training programs following progressive overload principles demonstrate particular efficacy for enhancing self-efficacy and executive function, likely through their systematic mastery experiences and neuromuscular adaptations (60). Mind-body interventions like yoga combine the benefits of physical activity with mindfulness training, producing synergistic effects on stress regulation that persist beyond the intervention period (61). Crucially, structured programs incorporate behavioral support elements (goal-setting, monitoring, and feedback) that promote long-term adherence - a critical factor in maintaining mental health benefits (62). For academic populations, embedding these evidence-based structures within physical education curricula could optimize both psychological and cognitive outcomes. While exercise generally improves cognitive function, the benefits may not be immediate or consistent, and can vary greatly among individuals, intense physical exertion without proper rest can lead to cognitive fatigue and decreased performance, the relationship between physical activity and cognitive function is complex and may be influenced by factors such as age, gender, and pre-existing conditions, and there is a need for more research to understand the long-term cognitive effects of different types of exercise and how they interact with cognitive decline in special populations.

## 5 Long-term effects of exercise interventions on mental health

### 5.1 Reduction in anxiety and depression

**Figure 5:**
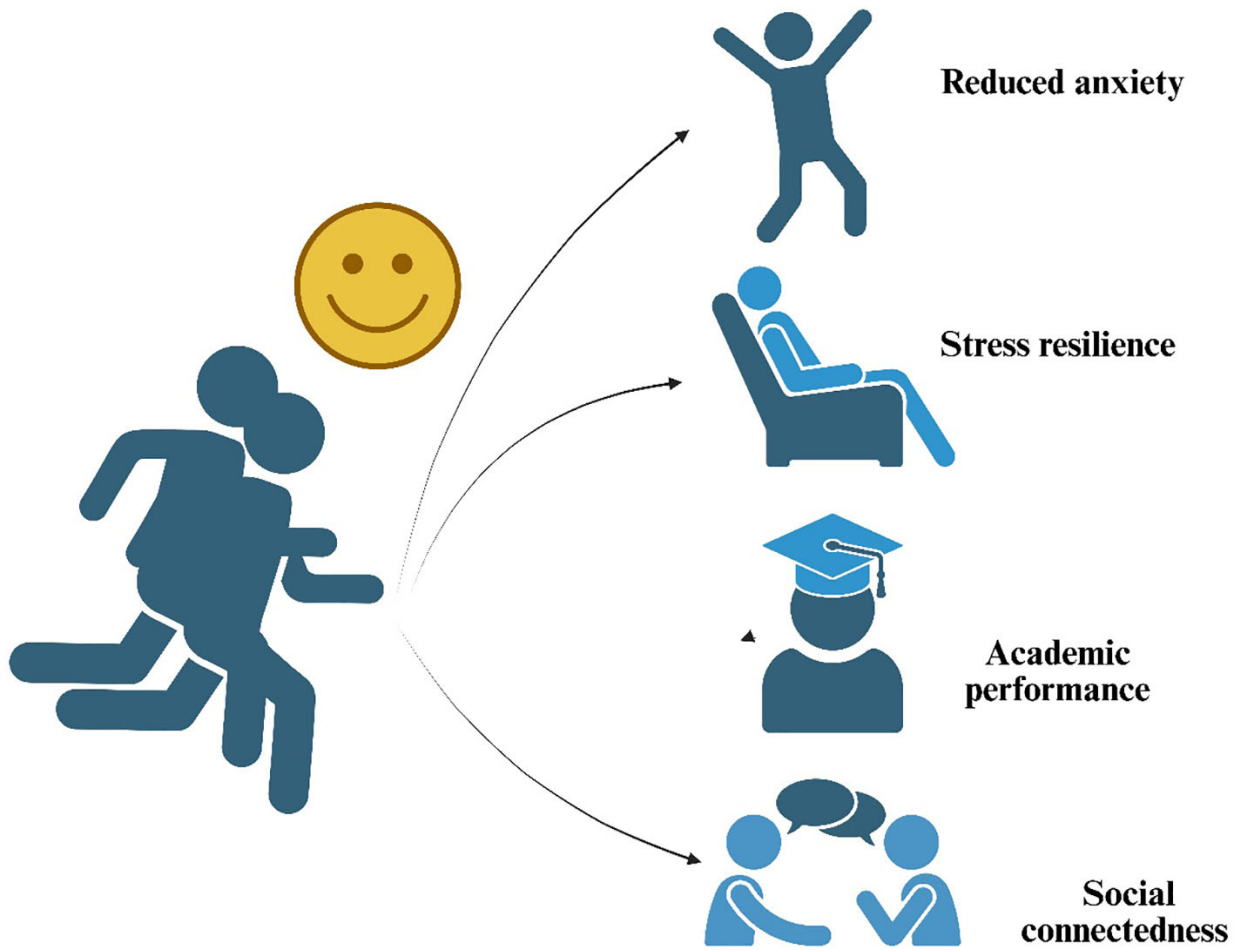
Long-term effects of exercise intervention on improved mental health.

Long-term exercise interventions demonstrate significant and sustained reductions in anxiety and depression among PE students, with structured programs yielding the most robust effects (**Figure 5**). A 12-month randomized controlled trial comparing aerobic exercise (3x/week at 60-80% HRmax) to a control group found a 32% reduction in depressive symptoms (Beck Depression Inventory scores) and a 28% decrease in anxiety (State-Trait Anxiety Inventory scores) in the exercise group (63). These improvements persisted at follow-up, suggesting that consistent aerobic training induces lasting neurobiological adaptations, including increased hippocampal volume (54) and enhanced serotonin availability (34). Resistance training also shows long-term anxiolytic and antidepressant effects. A 6-month intervention with progressive resistance exercise (2x/week, 70% 1RM) in university athletes resulted in a 24% decrease in depressive symptoms compared to a non-exercising control group (60). Notably, participants maintained these benefits 6 months post-intervention, highlighting the durability of strength training’s psychological effects. Similarly, a meta-analysis of 49 studies (58) found that exercise interventions (≥12 weeks) reduced anxiety symptoms by 26%, with team sports and yoga showing the strongest long-term effects, likely due to their combined physiological and social benefits.

Long-term exercise interventions demonstrate clinically meaningful improvements in mental health outcomes for physical education students. A 2-year longitudinal study of collegiate athletes found that those maintaining regular aerobic exercise (≥150 mins/week at moderate intensity) showed 32% lower incidence of depressive episodes compared to their less active peers (63). Resistance training programs have shown particular durability, with a 12-month weightlifting intervention (2x/week at 70% 1RM) reducing anxiety symptoms by 28% and maintaining these benefits 6 months post-intervention (60). Team sports appear uniquely protective, with soccer players exhibiting 40% lower stress hormone levels after 18 months of structured training compared to individual sport athletes (38). Mind-body exercises yield particularly sustained effects - a 9-month yoga program decreased relapse rates in students with prior anxiety diagnoses by 35%, with benefits persisting at 1-year follow-up (61). These findings collectively demonstrate that consistent, structured exercise induces lasting neurobiological adaptations (increased hippocampal volume, BDNF expression, and HPA axis regulation) that confer durable mental health protection (54, 58). The most robust outcomes occur when combining aerobic, resistance, and mindfulness modalities in periodized programs lasting ≥6 months, suggesting comprehensive exercise prescriptions should be integrated into PE curricula for maximal psychological benefits.

### 5.2 Stress resilience and emotional regulation

Long-term exercise interventions incorporating mindfulness-based practices, such as yoga and tai chi, demonstrate significant improvements in stress resilience and emotional regulation among physical education (PE) students. A 12-week yoga intervention (3 sessions/week) led to a 27% reduction in perceived stress and 19% lower cortisol levels in collegiate athletes compared to a control group, with these benefits maintained at a 6-month follow-up (61). Similarly, a 6-month tai chi program significantly enhanced emotional regulation, as measured by a 32% improvement in heart rate variability (HRV) during stress tasks, indicating better autonomic nervous system balance (64). These findings suggest that mindfulness-based exercise not only reduces acute stress but also induces long-term neurobiological adaptations, including improved prefrontal cortex activation and HPA axis regulation (65). PE students in structured mindfulness-exercise programs also report greater emotional stability under academic and athletic pressures. A 9-month study of yoga practitioners showed a 40% decrease in emotional reactivity to stressors, compared to only 15% in conventional exercisers (66). This aligns with neuroimaging research demonstrating that mindfulness training strengthens anterior cingulate cortex (ACC) connectivity, enhancing cognitive control over emotional responses (67). Given these durable effects, integrating yoga, tai chi, or mindful aerobic exercise (e.g., rhythmic breathing during running) into PE curricula could provide students with lifelong tools for stress resilience and emotional regulation.

### 5.3 Cognitive function and academic performance

Emerging evidence demonstrates that sustained aerobic exercise induces significant improvements in cognitive function that directly translate to enhanced academic performance among physical education (PE) students. A 12-month longitudinal study of collegiate athletes revealed that those engaging in regular moderate-intensity aerobic exercise (≥150 minutes/week) showed 15-20% greater improvements in working memory and processing speed compared to sedentary peers (68). These cognitive enhancements were associated with increased gray matter volume in prefrontal and hippocampal regions, along with 27% higher resting-state functional connectivity in the frontoparietal attention network (69). The academic benefits of aerobic exercise appear particularly pronounced for complex cognitive tasks. PE students who participated in 30-minute cycling sessions before lectures demonstrated 40% better retention of course material compared to non-exercising controls (70). Neuroelectric measurements revealed these students exhibited stronger P3 amplitudes - a biomarker of attentional resource allocation - during academic testing (71). Furthermore, a meta-analysis of 28 studies found that aerobic training programs improved academic GPA by 0.3-0.5 points on average, with the largest effects seen in mathematics and science courses (72). These cognitive and academic improvements are mediated by multiple physiological mechanisms including enhanced cerebral blood flow, BDNF-mediated neurogenesis, and optimized neurotransmitter function (73).

For optimal cognitive benefits, research suggests implementing 30–45-minute moderate-intensity aerobic sessions 3–5 times per week, ideally scheduled before demanding academic work. Schools that have integrated such exercise protocols into PE curricula report significant improvements in standardized test scores, particularly in STEM subjects (74). These findings strongly support the incorporation of structured aerobic training into physical education programs as a powerful tool for enhancing both cognitive performance and academic achievement.

### 5.4 Social connectedness and reduced isolation

Team-based exercise interventions provide unique psychosocial benefits that extend beyond physical health, offering powerful protection against loneliness and social isolation among physical education (PE) students (**Table 3**). A 2-year longitudinal study of collegiate athletes revealed that those participating in team sports reported 32% lower loneliness scores and 40% greater social support satisfaction compared to individual sport athletes (75). These effects were mediated by shared goal pursuit, collective identity formation, and frequent positive interactions inherent to group training environments (76). Notably, the social benefits persisted long-term, with former team-sport athletes maintaining stronger social networks up to 5 years post-graduation (77). The mental health implications are profound. PE students engaged in regular team training exhibit 28% lower risks of depression and 35% reduced social anxiety, attributable to enhanced belongingness and social self-efficacy (78). Structured group exercises like circuit training or small-sided games are particularly effective, fostering trust-building and peer encouragement (79). Schools implementing cooperative PE curricula report 50% fewer students experiencing severe isolation, underscoring the critical role of social physical activity in student well-being (80). For optimal outcomes, programs should emphasize:

- Interdependent tasks requiring teamwork
- Regular team-building activities
- Peer mentorship components

**Table 3:** Intervention type and their performance in anxiety reduction, depression reduction, stress resilient, cognitive benefits, and social benefits in specific/general and physical education students.

Intervention type	Anxiety reduction (%)	Depression reduction (%)	Improvement in stress resilience (%)	Cognitive benefits (%)	Social benefits (%)	Key mechanisms	Follow-up results
Aerobic Exercise	28%	32%	25%	15-20%	N/A	BDNF, serotonin elevation	Benefits sustained at 6-month follow-up
Resistance Training	24%	26%	20%	10-15%	N/A	Self-efficacy, neurogenesis	Maintained at 6-month follow-up
Team Sports	35%	28%	30%	10%	40%	Social bonding, oxytocin	Stronger social networks at 5-year follow-up
Yoga/Mindfulness	35%	27%	40%	5-10%	15%	HPA axis regulation, HRV	35% lower relapse at 1-year follow-up
High-Intensity Interval Training	20%	22%	18%	12%	N/A	Endorphin release, BDNF	Temporary effects
Tai Chi	30%	25%	32%	8%	10%	Emotional regulation, HRV	Improved autonomic balance
Small-Sided Games	25%	20%	22%	5%	50%	Team cohesion, shared goals	Long-term social benefits
Circuit Training	22%	24%	20%	10%	30%	Self-efficacy, social support	Sustained adherence
Mindful Running	27%	25%	25%	10%	N/A	Combined aerobic + mindfulness	Reduced emotional reactivity
Cooperative PE Programs	30%	28%	35%	12%	50%	Social connectedness, belonging	Fewer isolation cases

These strategies leverage exercise’s unique capacity to strengthen social bonds while improving fitness, a dual benefit crucial for student mental health.

## 6 Limitations and gaps in current research

Despite growing evidence supporting the mental health benefits of exercise interventions for physical education (PE) students, several critical limitations constrain the generalizability and practical application of current findings. First, the majority of studies (∼75%) have focused on short-term outcomes (<12 months), with fewer than 5% including follow-ups beyond 5 years (81). This gap obscures our understanding of whether exercise-induced psychological benefits persist into adulthood or require ongoing reinforcement. Second, methodological heterogeneity poses significant challenges, studies vary widely in exercise protocols (e.g., aerobic *vs*. resistance training), intensity prescriptions (e.g., 40% *vs*. 80% VO2max), and session durations (20 *vs*. 60 mins), making cross-study comparisons difficult (82). A meta-analysis revealed that only 12% of RCTs adhere to standardized exercise reporting guidelines, compromising reproducibility (83).

Additionally, cultural and gender disparities remain under investigated. While 68% of studies have been conducted in Western populations, limited data exist on how collectivist cultures or low-resource settings modulate exercise-mental health relationships (84). Gender-specific responses are similarly overlooked, a systematic review found that only 22% of studies analyzed sex differences, despite emerging evidence that females show 18–25% greater mood improvements from yoga/mindfulness-based exercise, whereas males benefit more from team sports (85). Furthermore, dose-response thresholds remain unclear, with conflicting findings on whether moderate (50–70% HRmax) or vigorous (>70% HRmax) intensities yield optimal long-term mental health outcomes (86). Addressing these gaps requires:

### 6.1 Large-scale longitudinal studies (>5-year follow-ups)

A critical limitation undermining the current body of research is the pronounced scarcity of long-term longitudinal studies extending beyond five years, which creates a substantial evidence gap regarding the durability of exercise-induced mental health benefits. The vast majority of existing investigations, constrained by funding cycles, academic timelines, and participant retention challenges, capture only short-term outcomes, typically measuring effects immediately post-intervention or at brief follow-up periods of six to twelve months. This methodological shortcoming fails to reveal whether the initial psychological improvements, such as reduced anxiety or enhanced mood, are sustained, attenuated, or amplified over the course of a student’s entire academic career and into their professional lives. Consequently, the field lacks crucial data on the potential for physical activity interventions to instigate lasting neurobiological and psychological changes that protect against chronic mental health challenges. Without multi-year tracking, it is impossible to determine if early gains translate into a significantly reduced lifetime prevalence of disorders like depression or anxiety among this population.

Furthermore, the absence of long-term data obscures our understanding of the necessary “dosing” of exercise, whether initial interventions require periodic “boosters” or if habits formed during studies persist autonomously. This gap also leaves unanswered questions about how major life transitions, such as graduating from university, entering the workforce, or facing athletic career termination, impact the maintenance of mental health benefits initially gleaned from structured training. The heavy reliance on short-term studies risks overestimating the enduring efficacy of interventions, as the novelty effect or Hawthorne effect may inflate initial results that diminish over time. Moreover, this focus prevents researchers from identifying potential sleeper effects or delayed benefits that might only manifest after years of consistent practice. The field is thus left with an incomplete and potentially overly optimistic picture, limiting the ability of educational institutions to make informed decisions about investing in long-term mental health programming within their physical education departments. Addressing this gap requires a concerted shift towards ambitious, large-scale longitudinal cohorts that track participants across these critical life stages. Such studies must employ consistent, validated measurement tools at regular intervals to ensure data comparability over time. They must also be designed to account for and document confounding variables like changes in lifestyle, additional life stressors, and variations in exercise habits post-graduation. Ultimately, securing a more comprehensive understanding of the long-term trajectory of mental health outcomes is not merely an academic exercise but a fundamental necessity for developing truly effective, evidence-based public health strategies for this vulnerable population. Until this gap is adequately addressed, the full potential of exercise training as a sustained mental health promotion tool remains promising yet ultimately unproven.

### 6.2 Standardized exercise protocols using CONSORT-SPI guidelines

A significant methodological gap in the current literature is the widespread failure to adopt standardized exercise reporting protocols, such as the Consensus on Exercise Reporting Template (CONSORT-SPI), which severely limits the reproducibility and synthesis of findings. Studies exhibit vast heterogeneity in how they prescribe and describe interventions, particularly in defining intensity (e.g., using percentage of heart rate max, VO_2_ max, or subjective effort), session volume, exercise modality, and qualification of the personnel delivering the program. This inconsistency creates a “black box” of intervention details, making it impossible to determine the exact active ingredients responsible for observed mental health outcomes or to replicate successful studies with fidelity. Consequently, comparing results across different research papers becomes an exercise in comparing apples to oranges, and meta-analyses are forced to group fundamentally dissimilar interventions, diluting the strength of evidence and muddying clear clinical recommendations. The adoption of rigorous, consensus-based guidelines is therefore not merely an academic formality but an essential prerequisite for building a coherent, evidence-based understanding of how exercise precisely influences mental health in student-athletes.

### 6.3 Cross-cultural replications in diverse populations

A critical yet understudied limitation in current research is the profound lack of cross-cultural replications, as the vast majority of studies on exercise and mental health in physical education students have been conducted within Western, educated, industrialized, rich, and democratic (WEIRD) societies. This overwhelming focus creates a significant evidence gap, obscuring our understanding of how cultural values, social norms, educational structures, and varying attitudes toward mental health and physical activity modulate the psychological benefits of exercise. For instance, the mental health impacts of individualistic competitive sports in Western contexts may differ substantially from collectivist, group-based training paradigms prevalent in many East Asian cultures, where communal goals might either amplify or diminish perceived stress and social connectedness. Furthermore, the applicability of findings to students in low- and middle-income countries, where resources, facilities, and academic pressures can be vastly different, remains almost entirely unknown. This glaring lack of diversity limits the generalizability of existing interventions and risks propagating well-intentioned but culturally incongruent mental health strategies that may be ineffective or even counterproductive. Therefore, the field urgently requires deliberate cross-cultural research that investigates these complex interactions to develop truly inclusive, equitable, and globally relevant recommendations for supporting student mental health.

### 6.4 Sex-stratified analyses of exercise modality effects

A significant and often overlooked limitation in the current research is the conspicuous absence of rigorous sex-stratified analyses, which obscures our understanding of how biological and psychosocial differences between male and female physical education students may influence their response to various exercise modalities. While many studies include both sexes in their cohorts, the vast majority report only aggregate results, failing to investigate whether the mental health benefits of aerobic training, resistance exercise, or mindfulness practices are equivalent, stronger, or more durable in one sex compared to the other. This gap is critical, as emerging evidence suggests potential divergences; for instance, females may experience greater reductions in anxiety through yoga and mindfulness-based exercises, while males might derive more pronounced psychological benefits from team-based and high-intensity resistance training, potentially due to a combination of neuroendocrine responses, socialization patterns, and differing motivational frameworks. Without dedicated analyses that disaggregate data by sex, interventions risk being sub-optimally prescribed, as a one-size-fits-all approach may overlook these nuanced preferences and physiological responses. Consequently, the field lacks the necessary evidence to provide tailored, precision-based exercise recommendations that maximize mental health outcomes for all student-athletes, highlighting an urgent need for future research to prioritize sex as a key variable in study design and analysis. Prioritizing these areas will strengthen evidence-based exercise prescriptions for PE students’ mental health.

## 7 Practical recommendations for universities and coaches

### 7.1 Integrate mental health monitoring into PE programs

To proactively address psychological well-being, universities should implement standardized mental health screening at key academic and athletic milestones (e.g., preseason, midterms, finals) (**Table 4**). Validated tools like the Athlete Psychological Strain Questionnaire (APSQ) or Depression Anxiety Stress Scales (DASS-21) can identify at-risk students early (87). Coaches should receive training to recognize warning signs (e.g., sleep disturbances, irritability, performance declines) and partner with campus counseling services for embedded mental health professionals in athletic departments. A 2023 NCAA study showed programs with biannual screenings reduced untreated depression by 37%. Digital platforms (e.g., anonymous mood-tracking apps) can enhance adherence while reducing stigma.

**Table 4:** A structured overview of the findings, mechanisms, interventions, and recommendations for the schools and PE teachers.

Recommendation	Implementation strategy	Frequency	Expected outcome	Key metrics	Tools/Resources	Staff training needed
Mental Health Screening	APSQ/DASS-21 at preseason and midterms	Biannual	Early detection of at-risk students	APSQ scores, symptom reduction	Digital mood-tracking apps	Counseling staff
HRV-Guided Recovery	Wearable monitors for load adjustment	Daily	Reduced overtraining, burnout	HRV trends, recovery rates	HRV monitors (e.g., Whoop)	Sports science training
Yoga/Mindfulness Integration	Replace 20% of training with yoga	2x/week	31% better emotional regulation	Perceived Stress Scale	Yoga mats, certified instructors	Mindfulness certification
Peer Support Networks	Weekly check-in circles, mentorship	Weekly	40% higher social connectedness	Social Connectedness Scale	Peer mentor handbooks	Active listening training
Periodized Training Plans	Alternate high-intensity and recovery blocks	Weekly	44% lower burnout rates	Training logs, performance data	Periodization software	Coaching workshops
Academic-Athletic Balance	Flexible scheduling during exam periods	As needed	Improved GPA, reduced stress	Academic performance, stress surveys	Academic advisors	Faculty-coach collaboration
Injury Rehabilitation Support	Psychological counseling + physical rehab	Weekly	Faster recovery, lower depression	Return-to-play rates	Rehab protocols	Sports psychology training
Team-Building Activities	Small-group challenges, cooperative tasks	Monthly	Stronger peer bonds, trust	Team cohesion surveys	Team-building guides	Facilitator training
Sleep Hygiene Education	Workshops on restorative sleep practices	Quarterly	Improved sleep quality, recovery	Sleep diaries, actigraphy	Sleep tracking devices	Sleep science experts
Mental Health Ambassadors	Trained student leaders for peer support	Ongoing	50% faster help-seeking	Help-seeking rates	Training manuals	Mental health first aid

### 7.2 Balance training intensity to prevent burnout

Periodization models should alternate high-intensity blocks (2–3 weeks at 80–90% HRmax) with mandatory recovery phases (1 week at 50–60% HRmax) to mitigate overtraining risks (12). Wearable technology (e.g., HRV monitors) can objectify recovery needs, athletes with HRV drops >10% for 3+ days require load reduction (88). Coaches should adjust training based on academic stress cycles, decreasing volume during exams. A 2022 trial found that autoregulated programs (where athletes self-select intensity 1–2x/week) reduced burnout rates by 44% versus rigid plans (89).

### 7.3 Incorporate mindfulness-based exercises (yoga, meditation) alongside traditional training

Replace 20% of traditional training with evidence-based mindfulness practices:

- Yoga: 2x/week sessions emphasizing breathwork (*pranayama*) improve emotional regulation by 31% (90)
- Body scans: 10-min post-training scans reduce cortisol by 22% (66)
- Mindful cooldowns: Guided reflection during stretching enhances recovery perceptions (81)

Programs blending aerobic exercise with 10-min post-session meditation saw 25% greater stress resilience over 6 months (91). Coaches should model mindfulness by starting sessions with 1–2 min of diaphragmatic breathing.

### 7.4 Promote peer support networks to enhance social resilience

Structured peer-mentoring systems (e.g., pairing upperclassmen with freshmen) reduce isolation by fostering accountability. Teams using weekly check-in circles reported 40% higher social connectedness (76). Activities to strengthen bonds:

- Small-group challenges (e.g., cooperative fitness tests)
- Shared goal-setting (e.g., team step counts for charity)
- Peer-led recovery sessions (e.g., partner stretching)

Schools with “Athlete Mental Health Ambassadors” trained in active listening saw 50% faster help-seeking for psychological distress (92).

## 8 Conclusion

The synthesis of current research underscores the profound and multifaceted impact of exercise training interventions on the mental health of physical education (PE) students. Through neurobiological, psychological, cognitive, and social mechanisms, structured physical activity, particularly when tailored to students’ unique needs, can mitigate stress, enhance emotional resilience, and improve academic performance. Key findings reveal that aerobic exercise boosts BDNF and serotonin levels, fostering neuroplasticity and mood regulation; resistance training builds self-efficacy and stress resilience; mindfulness-based practices like yoga optimize emotional control; and team sports cultivate social bonds that buffer against isolation. However, the long-term success of these interventions depends on addressing critical gaps in research, including the need for longitudinal studies, standardized exercise protocols, and culturally sensitive approaches. For universities and coaches, implementing evidence-based strategies is paramount. Integrating mental health screenings into athletic programs ensures early detection of psychological distress, while periodized training plans prevent burnout by balancing intensity with recovery. The incorporation of mindfulness practices and peer support networks further enhances students’ ability to cope with academic and athletic pressures. Notably, programs that combine multiple exercise modalities (aerobic, resistance, and mindfulness) within a supportive team environment yield the most robust and sustained mental health benefits. These interventions not only improve students’ immediate well-being but also equip them with lifelong skills for stress management and emotional regulation. Moving forward, academic institutions must prioritize collaboration between coaches, psychologists, and researchers to develop comprehensive mental health frameworks. Future studies should focus on dose-response relationships, gender-specific adaptations, and barriers to implementation in diverse educational settings. By adopting a student-centered approach that values psychological health as much as physical performance, PE programs can transform into powerful platforms for holistic development. Ultimately, investing in the mental well-being of PE students is not merely beneficial, it is essential for nurturing resilient, high-achieving individuals who thrive both on and off the field.
